# Real-world efficacy and safety of golidocitinib in 3 patients with relapsed/refractory peripheral T-cell lymphoma

**DOI:** 10.3389/fonc.2026.1891177

**Published:** 2026-08-10

**Authors:** Bianhong Wang, Fan Yu, Yanying Wang, Xuehan Mao, Jiao Li, Jingxian Li, Zhengshuo Jin, Lihong Li, Yuehua Huang

**Affiliations:** Department of Hematology, Beijing Tsinghua Changgung Hospital, School of Clinical Medicine, Tsinghua Medicine, Tsinghua University, Beijing, China

**Keywords:** clinical efficacy, golidocitinib, real-world study, relapsed/refractory peripheral T-cell lymphoma, safety

## Abstract

**Objective:**

To evaluate the real-world efficacy and safety of golidocitinib in patients with relapsed/refractory peripheral T-cell lymphoma (R/R PTCL).

**Methods:**

Clinical data of 3 patients with R/R PTCL treated with golidocitinib at our center between May 2024 and November 2025 were retrospectively analyzed. Baseline features, treatment regimens, responses, and adverse events (AEs) were collected and assessed.

**Results:**

Pathological subtypes included PTCL-not otherwise specified (PTCL-NOS, n=2) and monomorphic epitheliotropic intestinal T-cell lymphoma (MEITL, n=1). Following golidocitinib-based therapy, 2 patients achieved complete response (CR) and 1 partial response (PR), yielding an objective response rate (ORR) of 100%. Median follow-up was 6.2 months (range: 4.2–9.1 months); median progression-free survival (PFS) was not reached. Treatment-related AEs were predominantly hematological, mostly grade 1–2 and manageable. No treatment-related deaths occurred.

**Conclusion:**

Golidocitinib exhibits promising efficacy and a tolerable safety profile in R/R PTCL, including rare subtypes, supporting its role as a valuable therapeutic option. Larger cohorts are needed for further validation.

## 1 Introduction

Peripheral T-cell lymphoma (PTCL) represents a heterogeneous and aggressive subgroup of non-Hodgkin lymphoma (NHL) derived from mature T lymphocytes and natural killer (NK) cells (1). Its epidemiology shows marked geographic disparity, with a substantially higher incidence in Asian populations. In China, PTCL accounts for approximately 25.6% of all NHL cases, a proportion several times higher than that reported in Western countries, where the prevalence ranges from 5% to 10% (2, 3).

Most PTCL subtypes, including PTCL-not otherwise specified (PTCL-NOS), angioimmunoblastic T-cell lymphoma (AITL), and monomorphic epitheliotropic intestinal T-cell lymphoma (MEITL), exhibit aggressive clinical behavior and poor long-term prognosis (4, 5). Standard first-line therapy relies on anthracycline-based chemotherapy, followed by autologous stem cell transplantation (ASCT) in eligible patients (6). However, the majority of patients eventually develop relapsed or refractory (R/R) disease, and effective salvage options remain limited, representing a major unmet clinical need (7, 8).

Persistent activation of the JAK-STAT signaling pathway is a hallmark of PTCL pathogenesis, driving malignant proliferation, survival, and immune evasion (9–11). Targeting this pathway has emerged as a promising therapeutic strategy. Golidocitinib, a novel highly selective JAK1 inhibitor, specifically disrupts JAK1-mediated STAT signaling with minimal off-target inhibition of JAK2, JAK3, or TYK2 (12). Phase 2 clinical trials have demonstrated robust antitumor activity and a favorable safety profile in patients with R/R PTCL (12). Real-world evidence, however, remains limited. This retrospective case series aimed to evaluate the clinical efficacy and safety of golidocitinib-based therapy in three patients with R/R PTCL treated in routine clinical practice.

## 2 Patients and methods

### 2.1 Study population

Three patients diagnosed with R/R PTCL and treated with golidocitinib between May 2024 and November 2025 were enrolled.

#### 2.1.1 Inclusion criteria

1. Pathologically confirmed PTCL by biopsy and immunohistochemistry according to the WHO classification of hematolymphoid tumors;
2. At least one line of prior systemic therapy;
3. Relapsed or refractory disease after first-line chemotherapy;
4. At least 2 cycles of golidocitinib monotherapy or combination therapy;
5. Written informed consent for golidocitinib treatment.

#### 2.1.2 Exclusion criteria

1. Severe cardiac, hepatic, or renal dysfunction;
2. Uncontrolled active infection;
3. Expected survival <3 months;
4. Pregnancy or lactation.

### 2.2 Treatment regimen

Golidocitinib was administered orally at 150 mg once daily until disease progression or unacceptable toxicity. Combination regimens included:

- CMOP (liposomal mitoxantrone + vindesine + cyclophosphamide + prednisone);
- CHOPE (etoposide + cyclophosphamide + vindesine + epirubicin + prednisone);
- MGDP (liposomal mitoxantrone + gemcitabine + cisplatin + dexamethasone).

Dose adjustments were made based on toxicity. Routine laboratory tests including complete blood count and liver/renal function were monitored during treatment.

### 2.3 Assessment of efficacy and safety

Treatment response was evaluated by PET/CT and contrast-enhanced CT according to the Lugano criteria: complete response (CR), partial response (PR), stable disease (SD), and progressive disease (PD). Objective response rate (ORR) was defined as CR + PR. Disease control rate (DCR) was defined as CR + PR + SD. Progression-free survival (PFS) was calculated from the initiation of golidocitinib to disease progression or death. Safety was assessed using the National Cancer Institute Common Terminology Criteria for Adverse Events (CTCAE) version 5.0.

CMV and EBV viral loads were quantified by plasma real-time quantitative PCR with a lower detection limit of 4.0×10² copies/mL. PCR tests were ordered only when clinical signs of infection emerged. Archived records contained complete CMV-DNA data but no systematic CMV IgG/IgM serology. We acknowledge periodic combined serology and PCR screening is recommended for future cohorts, which is noted as a limitation in Discussion.

### 2.4 Statistical analysis

Descriptive statistical analysis was performed. Categorical data were presented as number (percentage). Continuous data were expressed as median (range). Survival data were analyzed descriptively; median follow-up and median PFS were reported as median (range).

### 2.5 Ethical approval

This retrospective study involving human participants was reviewed and approved by the Ethics Committee of Beijing Tsinghua Changgung Hospital, in accordance with the Declaration of Helsinki and the local legislation and institutional requirements. Written informed consent for study participation was waived by the ethics committee due to the anonymized nature of the analyzed data and the minimal risk to participants. Written informed consent for publication was obtained from all participants for the publication of any potentially identifiable images or data included in this article.

## 3 Results

### 3.1 Baseline characteristics

Among the 3 patients, 2 were male and 1 was female, with a median age of 71 years (range 58–88 years). Two patients had PTCL-NOS and one had MEITL. All patients were relapsed or refractory after first-line chemotherapy. One patient received mini-CHOP followed by CMOP; two patients received CHOPE, one of whom also received an EZH2 inhibitor. ECOG performance status was 0 in 1 patient and 1 in 2 patients (**Table 1**).

**Table 1:** Baseline characteristics of 3 patients with R/R PTCL.

Case	Age/Gender	Pathological type	Stage	Blood routine	Bone marrow	Genetic features	Prior chemotherapy	Efficacy and outcome
1	88/M	PTCL-NOS	IV	WBC 37.53×10^9^/L,Hgb 89g/L,PLT 17×10^9^/L	Abnormal lymphocytes 82.75%	TP53 mutation, complex karyotype	mini-CHOP×2 → golidocitinib + CMOP ×2	PR, death due to COVID-19 (unrelated to treatment/disease)
2	71/M	PTCL-NOS	IV	WBC 1.98×10^9^/L,Hgb 96g/L,PLT 55×10^9^/L	Abnormal lymphocytes 6%	DNMT3A, TET2, EZH2 mutations; normal karyotype	CHOPE ×6 → CHOPE + golidocitinib×2 → golidocitinib maintenance	CR, alive, ongoing remission
3	58/F	MEITL	II	WBC 7.29×10^9^/L,Hgb 116g/L,PLT 274×10^9^/L	Normal	No mutation testing; normal karyotype	CHOPE ×2 → CHOPE + EZH2 inhibitor ×1 → MGDP + golidocitinib ×3	PR, ongoing treatment, bridge to ASCT

### 3.2 Treatment response and survival follow-up

#### 2.2.1 Case 1

An 88-year-old male presented with dyspnea. PET/CT showed multiple hypermetabolic lymphadenopathy and splenomegaly. Bone marrow examination revealed 82.75% abnormal lymphocytes. He was diagnosed with PTCL-NOS stage IV, TP53 mutation, complex karyotype (very high risk). After 2 cycles of mini-CHOP (PD), he received golidocitinib plus CMOP for 2 cycles and achieved PR. He developed grade 4 myelosuppression, which was reversed by supportive care. He died of COVID-19 infection, unrelated to disease progression or treatment. Baseline CMV-DNA via real-time quantitative PCR was below the detection limit, and remained negative throughout treatment. Transiently elevated EBV-DNA (1.91×10^4^ copies/mL) was detected after COVID-19 infection, without CMV reactivation or related symptoms.

#### 2.2.2 Case 2

A 71-year-old male presented with abdominal pain, B symptoms, and lymphadenopathy. He was diagnosed with PTCL-NOS stage IV. After 6 cycles of CHOPE (SD, refractory), he received golidocitinib plus CHOPE for 2 cycles and achieved CR. He had grade 3 myelosuppression, which was manageable. He declined autologous stem cell transplantation due to financial constraints and has been on maintenance golidocitinib 150 mg qd since October 2025 with ongoing remission. Baseline and serial follow-up CMV-DNA measured by real-time quantitative PCR were persistently negative, with no clinical manifestations of CMV infection.

#### 2.2.3 Case 3

A 58-year-old female presented with chronic diarrhea. She was diagnosed with MEITL stage II. After 2 cycles of CHOPE and 1 cycle of CHOPE plus EZH2 inhibitor (PD), she received MGDP plus golidocitinib for 3 cycles and achieved PR. She developed grade 4 myelosuppression, which was reversed. She successfully collected CD34^+^ cells (8.47×10^6^/kg) and is planned for ASCT followed by golidocitinib maintenance. Pretreatment CMV-DNA was undetectable. Two follow-up PCR tests (August 16, 2025; March 17, 2026) remained negative, and no CMV reactivation was observed.

### 3.3 Overall efficacy

The ORR was 100% (3/3), with a CR rate of 66.7% (2/3). Median follow-up was 6.2 months (range 4.2–9.1 months). Median PFS was not reached; the 6-month PFS rate was 100% (3/3). Two patients successfully bridged to maintenance therapy or transplant preparation.

### 3.4 Safety

Treatment-emergent adverse events (TEAEs) were predominantly hematological (**Table 2**). Grade ≥3 TEAEs included neutropenia (2/3), thrombocytopenia (2/3), and anemia (1/3). Non-hematological AEs included mild elevated transaminase (1/3) and fatigue (2/3). No treatment-related deaths occurred. None of the three patients experienced CMV reactivation on serial PCR testing. Only Case 1 presented transient EBV elevation after COVID-19, and no anti-CMV antiviral treatment was administered to any patient.

**Table 2:** Treatment-related adverse events (n=3).

Adverse event	All grades, n (%)	Grade ≥3, n (%)	Management
Neutropenia	2(66.7)	2(66.7)	G-CSF support
Thrombocytopenia	2(66.7)	2(66.7)	TPO-RA,platelet transfusion
Anemia	2(66.7)	1(33.3)	Erythropoietin
Elevated transaminase	1(33.3)	0	Hepatoprotective agents
Fatigue	2(66.7)	0	Symptomatic treatment

## 4 Discussion

Patients with relapsed/refractory peripheral T-cell lymphoma (R/R PTCL) have a poor prognosis and limited standard treatment options. Aberrant activation of the JAK-STAT pathway is common across PTCL subtypes, driving tumor cell proliferation and survival (13, 14). Conventional chemotherapy lacks targeted activity against this pathway, resulting in unsatisfactory outcomes in the relapsed/refractory setting. Targeted inhibition of JAK signaling has therefore emerged as a promising therapeutic strategy for R/R PTCL. As a highly selective JAK1 inhibitor, golidocitinib directly suppresses tumor proliferation through the JAK1-STAT3 axis and modulates the tumor microenvironment to reverse T-cell exhaustion (15, 16). It acts synergistically with chemotherapy and other targeted agents (17–21) and exhibits a more favorable safety profile than non-selective JAK inhibitors, with reduced risks of anemia, thrombocytopenia, and immune-related disturbances (22).

In this case series, all three patients achieved clinical responses, with an overall response rate (ORR) of 100% and a complete response (CR) rate of 66.7%. The cohort included two patients with PTCL-not otherwise specified (PTCL-NOS) and one patient with rare monomorphic epitheliotropic intestinal T-cell lymphoma (MEITL). Case 1 represented an ultra-high-risk patient with advanced age, TP53 mutation, and complex karyotype, who still achieved meaningful clinical benefit. Case 3, diagnosed with MEITL-a subtype typically resistant to conventional chemotherapy-rapidly attained partial response (PR) with golidocitinib-based combination therapy, enabling successful stem cell collection and serving as a potential bridge to autologous stem cell transplantation (ASCT). These findings support the antitumor activity of golidocitinib in both common and rare PTCL subtypes and highlight its potential value as a transplant-bridging agent. For patients ineligible for transplantation, golidocitinib maintenance therapy provides durable disease control. The JACKPOT26 study demonstrated that golidocitinib maintenance yielded a 24-month disease-free survival (DFS) rate of 74.2% in CR patients and a median progression-free survival (PFS) of 17.4 months in PR patients (12). Notably, our only several-month maintenance follow-up cannot confirm the long-term durable disease-free survival benefit demonstrated in the 24-month data from the JACKPOT26 trial. Our small sample size and relatively short observation period restrict us from drawing definite conclusions on long-term survival outcomes. Consistently, our Case 2 maintained continuous remission with single-agent golidocitinib, which aligns with findings from previous clinical trials (12, 23). Treatment-related adverse events (TRAEs) were manageable in this study. Most adverse events were grade 1–2 hematological toxicities, and grade ≥3 events were reversible with supportive care. No treatment-related deaths occurred, which is consistent with the known safety profile of golidocitinib.

Several limitations should be acknowledged. This is a single-center, retrospective case series with a very small sample size (n=3), which limits generalizability and increases the risk of selection bias. The median follow-up duration is relatively short (6.2 months), so long-term efficacy, durability of response, and late adverse events cannot be fully evaluated. Additionally, the study lacks a control group and correlative biomarker analyses, such as JAK-STAT pathway activation status, minimal residual disease assessment, or genomic profiling, limiting our ability to identify predictors of response or resistance. Treatment regimens were heterogeneous, precluding definitive conclusions regarding the optimal combination strategy. Another limitation of this retrospective cohort was the absence of standardized routine CMV serology and pre-scheduled serial PCR surveillance, which limits comprehensive assessment of latent CMV reactivation risk. Combined serology and serial PCR monitoring are recommended for future prospective studies. Finally, one patient died of COVID-19 infection unrelated to disease progression or treatment, which may affect survival interpretation. Larger, prospective, multicenter studies with longer follow-up, comprehensive biomarker and virological monitoring are needed to validate these findings and further define the role of golidocitinib in routine clinical practice for patients with R/R PTCL.

## 5 Conclusion

Golidocitinib, a highly selective JAK1 inhibitor, demonstrates a high objective response rate, favorable disease control, and manageable safety in patients with R/R PTCL, including rare and high-risk subtypes. It represents a valuable clinical option for this underserved patient population.

## Data Availability

The data analyzed in this study is subject to the following licenses/restrictions: Written informed consent for publication was obtained from the participant(s). The dataset contains sensitive patient health information and is protected by institutional ethics and privacy regulations. It is not publicly available to ensure patient confidentiality. Access to anonymized data may be granted upon reasonable request to the corresponding author, subject to approval from the institutional review board and completion of a data use agreement. Requests to access these datasets should be directed to Yuehua Huang, hyha00833@btch.edu.cn.
